# Soluble Suppression of Tumorigenicity 2 (sST2) in Patients with Predominantly Decompensated Right Heart Failure—A Prospective Observational Study

**DOI:** 10.3390/jcm12237200

**Published:** 2023-11-21

**Authors:** Victoria Dworok, Valentin Hähnel, Marwin Bannehr, Vera Paar, Christoph Edlinger, Michael Lichtenauer, Christian Butter, Anja Haase-Fielitz

**Affiliations:** 1 Department of Cardiology, Heart Center Brandenburg Bernau & Faculty of Health Sciences (FGW) Brandenburg, Brandenburg Medical School (MHB) Theodor Fontane, Ladeburger Straße 17, 16321 Bernau bei Berlin, Germany; 2 Clinic of Internal Medicine II, Department of Cardiology, Paracelsus Medical University of Salzburg, 5020 Salzburg, Austria; 3 Institute of Social Medicine and Health System Research, Otto von Guericke University Magdeburg, 39120 Magdeburg, Germany

**Keywords:** Soluble Suppression of Tumorigenicity 2 (sST2), biomarker, NT-proBNP, decompensated heart failure

## Abstract

Right heart failure is a major challenge in clinical practice. Soluble Suppression of Tumorigenicity-2 (sST2), a member of the interleukin-1-receptor family, may have clinical prognostic value. The aim of this study was to analyze whether sST2 correlates with signs of acute right heart decompensation. This prospective single-center study included 50 patients admitted for clinical signs of predominant right heart decompensation. Signs of reduced blood supply to other organs (e.g., renal function parameter, troponin T, NT-proBNP), diuretics, and signs of venous congestion (inferior vena cava (IVC) diameter) with fluid retention (weight gain, peripheral edema) resulting from reduced RV function were analyzed. The degree of peripheral edema was defined as none, mild (5–6 mm depressible, regression in 15–60 s) or severe (>7 mm depressible, regression in 2–3 min). sST2 levels were measured at the day of hospitalization. A total of 78.7% showed severe peripheral edema. The median concentration of sST2 was 35.2 ng/mL (25.–75. percentiles 17.2–46.7). sST2 is correlated with the peripheral edema degree (rSpearman = 0.427, *p* = 0.004) and the diameter of IVC (r = 0.786, *p* = 0.036), while NT-proBNP (r = 0.114, *p* = 0.456), troponin T (r = 0.123, *p* = 0.430), creatinine-based eGFR (r = −0.207, *p* = 0.195), or cystatin C-based eGFR (r = −0.032, *p* = 0.839) did not. sST2, but no other established marker, is correlated with peripheral and central fluid status in patients with decompensated right heart failure.

## 1. Introduction

Acute heart failure (HF) is a major cause of hospitalization and mortality within Western countries [1]. Right heart failure is particularly associated with a poor prognosis, as both diagnosis and treatment remain a major challenge in clinical practice [2]. Natriuretic peptides such as NT-proBNP have been established as indispensable standard laboratory parameters for diagnosis, as well as for monitoring the efficacy of therapy and assessing prognosis in patients with chronic HF [3]. Although its value in clinical practice has been proven by numerous prospective studies, there are certain limitations, especially in acute and predominantly right HF [4,5]. Since natriuretic peptides are mainly secreted in the left atrium, the extent and severity of right cardiac diseases may not be adequately assessed by those markers.

Additionally, many patients with preexisting chronic HF also suffer from impaired renal function or cardiorenal syndrome, which could further reduce the validity of previously established biomarkers. In recent years, a variety of new cardiac biomarkers have been identified [6]. Soluble ST2 appears to be a promising biomarker for right HF and cardiorenal syndrome. As its predictive value may be less affected by renal function, it may allow an early diagnosis of subclinical HF and, consequently, a treatment of the prominent disease [7]. Soluble ST2 is a member of the interleukin-1 receptor family and plays a major role as a decoy receptor for interleukin-33. During the last few years, the marker has attracted much attention in the cardiovascular field since hemodynamic stress and cardiomyocyte burden have been identified as triggers of sST2 release [8]. Thus, sST2 has been shown to correlate with the prognosis in numerous cardiovascular diseases [9,10]. Moreover, sST2 has been included in the ACC/AHA guidelines for the management of acute and chronic HF, as a biomarker for better risk stratification [11].

The aim of this pilot study was to investigate the value of sST2 in patients with predominantly right HF. Our hypothesis was that sST2 might correlate with signs of decompensation in patients with predominantly right heart failure and, thus, could potentially be a valuable parameter to guide fluid management and diuretic dosing decisions.

## 2. Materials and Methods

### 2.1. Study Population and Design

In this single-center, prospective cohort study, we analyzed data from 50 adult patients admitted to the University Hospital Heart Centre Brandenburg for acute decompensated predominantly right HF and indication for loop diuretics therapy between October 2019 and October 2021. The Ethics Committee of the Brandenburg Medical School approved this study (E-01-20190603). 

### 2.2. Serum Biomarker Measurement

NT-proBNP, troponin-T, creatinine, cystatin C, and sST2 were determined at admission, the latter by a commercially available enzyme-linked immunosorbent assay (ELISA) kit (Human ST2/IL-33R DuoSet ELISA R&D Systems, Minneapolis, MN, USA). The Cobas e411 analyzer using the patented electrochemiluminescence technology for immunoassay analysis was used for NT-proBNP, troponin-T, and cystatin C measurement. Serum creatinine was measured by Beckman Coulter AU680 using the modified Jaffe method standardized by isotope dilution mass spectroscopy.

### 2.3. Data Collection

Medical records were reviewed and the following information was obtained: demographics, comorbidities, echocardiographic data, laboratory parameters, length of stay in hospital, rehospitalization within 90 days after discharge, and 90-day mortality. The estimated glomerular filtration rate (eGFR) was calculated using the Chronic Kidney Disease Epidemiology Collaboration (CKD-EPI) equation [12]. Follow-up was performed at three months for rehospitalization and mortality.

The primary endpoint of our study was the presence and degree of peripheral edema on clinical examination at the day of admission. We classified edema as follows: (1) no edema; (2) mild with 5–6 mm of depression and rebounding in 15–60 s or less; (3) severe edema with >7 mm of depression and rebounding in 2–3 min. 

In addition, laboratory parameters (such as NT-proBNP, cystatin C, NGAL, sST-2) were analyzed as secondary end points, possibly indicating early cardiorenal injury under diuretic use.

### 2.4. Statistical Analysis

Linear values are shown as median and IQR (25.–75. percentiles), and categorical variables are expressed as numeric values and percentages. Categorical variables were analyzed with Fisher’s exact or a chi-square test, and continuous variables with a nonparametric Mann–Whitney U-test and Kruskal–Wallis H-test. Spearman rank correlation was used to measure the degree of association between the degree of peripheral edema and the biomarker concentration at admission. Graphs were generated and data were analyzed using SPSS v27 (IBM, Armonk, NY, USA).

## 3. Results

### 3.1. Patient Characteristics

The baseline characteristics of the study subjects are presented in Table 1. The median age was 78.0 (70.0–83.0) years, and 48% of the patients were female. Chronic kidney disease was prevalent in 64% of patients, who were admitted with a median serum creatinine-based eGFR of 43 (33–60) mL/min/1.73 m^2^. Most patients showed severe (78%), some moderate (16%), and no (6%) peripheral edema, while 70.5% had albuminuria at the time of admission. Twenty percent of patients were readmitted within 90 days for decompensated heart failure; 24% of patients died within 90 days of index hospitalization.

### 3.2. Biomarker Results

**Figure 1:**
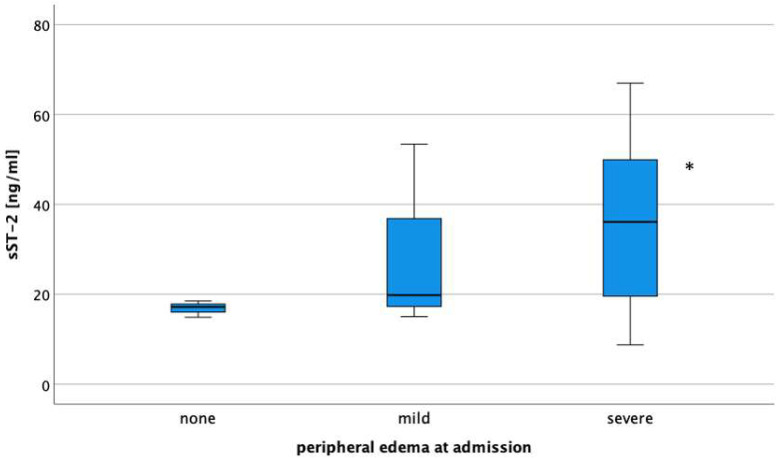
Median concentration of sST2 in patients with none, mild, and severe peripheral edema at the time of hospital admission. * *p* = 0.040.

**Figure 2:**
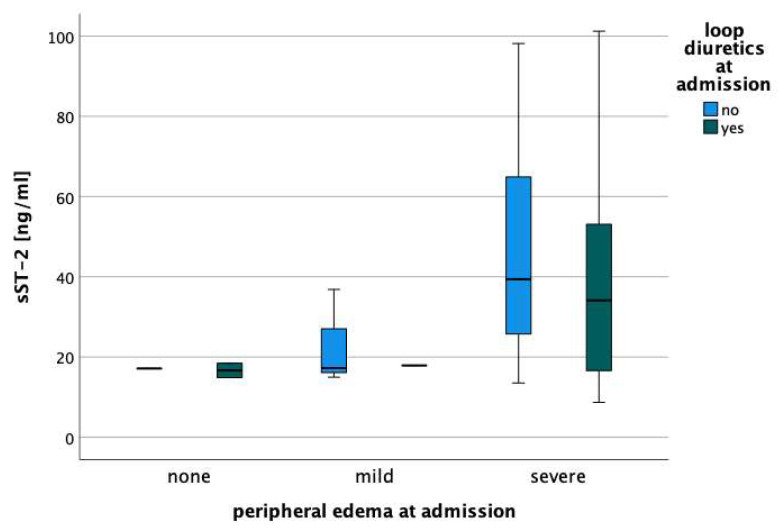
Median concentration of sST2 in patients with none, mild, and severe peripheral edema, in comparison with and without loop diuretics at the time of hospital admission.

Overall, median sST2 concentration at admission was 35.2 (17.2–46.7) ng/mL. sST2 concentrations differed depending on the severity of peripheral edema (*p* = 0.040; Figure 1). The pattern was essentially unchanged in patients already on loop diuretics at admission compared to those who were not (Figure 2).

Concentration of sST2 correlated with the degree of peripheral edema (rSpearman = 0.427, *p* = 0.004) and diameter of vena cava inferior (r = 0.786, *p* = 0.036), while biomarkers of renal function such as creatinine-based eGFR (r = −0.207, *p* = 0.195) and cystatin C-based eGFR (r = −0.032, *p* = 0.839), and established cardiac biomarkers, such as NT-proBNP (r = 0.114, *p* = 0.456) and troponin T (r = 0.123, *p* = 0.430), did not. 

## 4. Discussion

To the best of our knowledge, this is the first prospective study to analyze the value of sST2 as a biomarker for peripheral and central fluid assessment in patients hospitalized for predominantly right heart decompensation. 

Our findings can be summarized as follows:

(1) Levels of sST2 correlated with the degree of peripheral edema and vena cava width.
(2) Soluble ST2 was found to be superior to established biomarkers for cardiorenal diseases. Numerous prospective studies have shown that the predominantly right cardiac diseases are associated with a particularly poor prognosis, especially in those patients with concomitant cardiorenal syndrome [13,14].

Right heart failure is a complex disease with dysfunction of the right ventricular structures, which causes manifold issues and requires specific diagnostics. With improved imaging modalities and interventional approaches, the treatment os severe tricuspid regurgitation as a cause of right cardiac disease plays an increasingly important role in clinical practice [15]. In recent times, various minimally invasive transcatheter-based techniques have been established. Transcatheter edge to edge repair of the tricuspid valve has been introduced. Other procedures, such as the Tricvalve or Tricento procedure, are already in use but still in constant development [16].

However, other underlying causes for right heart failure, such as pulmonary hypertension, sarcoidosis, and amyloidosis, often remain challenging to diagnosis and treatment options [15].

Unfortunately, established biomarkers have their limitations in this setting, as they either correlate primarily with left cardiac disease, like natriuretic peptides, or have limited validity in the case of renal diseases [17,18].

Therefore, it would be of importance to have a biomarker available as an additional cornerstone for diagnosis, estimating the prognosis, but also as a follow-up parameter for monitoring and guidance for long-term treatment.

Compared to our study, AbouEzzeddine et al. found similarly high sST-2 levels in patients with preserved pump function, which showed a correlation with edema burden [9]. A correlation between elevated sST2 levels and comorbidities such as diabetes mellitus and atrial fibrillation could not be demonstrated in our cohort. Also, no sex differences in sST2 values were observed [9].

Our data indicate that sST2 could be a valuable link in clinical decision making on this issue, complementing the clinical impression. Moreover, in clinical practice, it often remains challenging, even for experienced physicians, to choose the right amount of loop diuretics in heart failure patients, who frequently show refractivity to diuretic drugs [5]. Our data suggest that sST2 correlates with clinical signs of acute decompensated heart failure in terms of peripheral edema and might therefore also be a valuable additional tool in short-term treatment. Moreover, the degree of decompensation in our cohort correlated with vena cava width. Although the pure measurement of vena cava width is not sufficient for an assessment of volume status, this parameter is included in innovative clinical tools such as the Vexus score [19]. In our opinion, it is conceivable that a combination of the Vexus score and one or more biomarkers such as sST2 could be used in the clinical assessment of fluid status in the future. However, whether sST2 is also valuable in long-term monitoring or in patients undergoing interventional treatments has to be investigated in further prospective studies.

Our data indicate that sST2 may provide additional information compared with natriuretic peptides. It has been described that sST2 is not as affected by the actual kidney function as NT-proBNP [20]. For this reason, we believe that sST2 could also be an important link in clinical practice in the very common constellation of right heart failure and cardiorenal syndrome.

### Limitations

Our study has some limitations. In particular, invasive measurement by right heart catheterization (i.e., pulmonary artery pressure, central venous pressure, right atrial pressure, and ventricular pressure) was not performed because of its invasiveness. This could be of interest in future studies, though. Another limitation of this study is the small sample size and the short follow-up period. However, as this is a pilot study, we are confident that the conclusions drawn have potential for prospective investigation in a substantially larger cohort. Further studies related to sST2 levels in patients with predominantly right cardiac decompensation are essential, because only monocentric prospective studies with a small number of cases have been performed so far.

## 5. Conclusions

Our data suggest that sST2, but no other established marker, is correlated with peripheral and central fluid status in patients with decompensated right heart failure. 

## Figures and Tables

**Table 1:** Baseline characteristics and outcome data.

Overall (N = 50)	
Age, years	78.0 (70.0–83.0)
Female, *n*	24 (48%)
Body weight	
At admission, kg	82.5 (69.8–101.9)
On day 3, kg	78.8 (67.5–93.2)
At discharge, kg	76.0 (57.5–90.5)
Cumulative diuretic dose i.v. day 1, mg	40 (40–80)
Cumulative diuretic dose day 1–3, mg	200 (120–340)
Severe peripheral edema at day of admission, *n*	39 (78%)
Ascites at day of admission, *n* *	6 (10.2%)
Auscultation findings	
Vesicular breathing sound	18 (36%)
Moist rales in lungs	22 (44%)
Basal attenuated breath sounds	10 (20%)
**Comorbidities**	
Arterial hypertension, *n* (%)	44 (88%)
Atrial fibrillation, *n* (%)	36 (72%)
Chronic kidney disease II-IV, *n* (%)	32 (64%)
Diabetes, *n* (%)	26 (52%)
Coronary heart disease, *n* (%)	25 (50%)
NYHA III, *n* (%)	32 (64%)
IV, *n* (%)	12 (24%)
Chronic obstructive pulmonary disease, *n* (%)	9 (18%)
Peripheral vascular disease, *n* (%)	5 (10%)
Left ventricular ejection fraction, %	40 (30–53)
Transtricuspid inflow maximum velocity	2.6 (2.14–4.9)
Systolic pulmonary arterial pressure (sPAP)	32.71 (18.31–73.61)
**Laboratory values at admission**	
sST2, ng/mL	35.2 (17.2–46.7)
eGFR at admission, ml/min/1.73 m^2^	43 (33–60)
Cystatin-C based eGFR, ml/min/1.73 m^2^	30 (20–44.5)
Albumin/serum creatinine ratio, g/mol	17.4 (6.7–54.6)
Albuminuria (>19 mg/L)	31 (70.5%)
Urine creatinine, µmol/L	4414 (3111–5647)
NT-proBNP, pg/mL	5256 (3331–9208)
Troponin T, pg/mL	40.7 (23.3–60.5)
**Outcome**	
Length of stay in hospital, days	8.5 (6–13.3)
Lung edema, *n*	2 (4%)
Acute kidney injury, *n*	8 (16%)
Rehospitalization within 90 days, *n*	10 (20%)
Died within 90 days, *n* (%)	12 (24%)

* Out of 44 patients.

## Data Availability

The data underlying this article will be shared upon reasonable request to the corresponding author.

## Abbreviations

sST2	Soluble Suppression of Tumorigenicity 2
HF	Heart failure
eGFR	Glomerular filtration rate
CKD-EPI	Chronic Kidney Disease Epidemiology Collaboration

## References

[1] Owan T.E., Hodge D.O., Herges R.M., Jacobsen S.J., Roger V.L., Redfield M.M. (2006). Trends in prevalence and outcome of heart failure with preserved ejection fraction. N. Engl. J. Med..

[2] Crisci G., D’Assante R., Valente V., Giardino F., D’Agostino A., Ranieri B., Arcopinto M., Marra A.M., Rainone C., Modestino M. (2023). How to Treat Right Heart Failure. Tips for Clinicians in Everyday Practice. Heart Fail. Clin..

[3] Feng J., Tian P., Liang L., Chen Y., Wang Y., Zhai M., Huang Y., Zhou Q., Zhao X., Zhao L. (2022). Outcome and prognostic value of N-terminal pro-brain natriuretic peptide and high-sensitivity C-reactive protein in mildly dilated cardiomyopathy vs. dilated cardiomyopathy. ESC Heart Fail..

[4] Werhahn S.M., Becker C., Mende M., Haarmann H., Nolte K., Laufs U., Zeynalova S., Löffler M., Dagres N., Husser D. (2022). NT-proBNP as a marker for atrial fibrillation and heart failure in four observational outpatient trials. ESC Heart Fail..

[5] Felker G.M., Ellison D.H., Mullens W., Cox Z.L., Testani J.M. (2020). Diuretic Therapy for Patients With Heart Failure: JACC State-of-the-Art Review. J. Am. Coll. Cardiol..

[6] Lichtenauer M., Jirak P., Wernly B., Paar V., Rohm I., Jung C., Schernthaner C., Kraus J., Motloch L.J., Yilmaz A. (2017). A comparative analysis of novel cardiovascular biomarkers in patients with chronic heart failure. Eur. J. Intern. Med..

[7] Bansal N., Zelnick L.R., Soliman E.Z., Anderson A., Christenson R., DeFilippi C., Deo R., Feldman H.I., He J., Ky B. (2021). Change in Cardiac Biomarkers and Risk of Incident Heart Failure and Atrial Fibrillation in CKD: The Chronic Renal Insufficiency Cohort (CRIC) Study. Am. J. Kidney Dis. Off. J. Natl. Kidney Found..

[8] Wernly B., Lichtenauer M., Jirak P., Eder S., Reiter C., Kammler J., Kypta A., Jung C., Franz M., Hoppe U.C. (2017). Soluble ST2 predicts 1-year outcome in patients undergoing transcatheter aortic valve implantation. Eur. J. Clin. Investig..

[9] AbouEzzeddine O.F., McKie P.M., Dunlay S.M., Stevens S.R., Felker G.M., Borlaug B.A., Chen H.H., Tracy R.P., Braunwald E., Redfield M.M. (2017). Suppression of Tumorigenicity 2 in Heart Failure With Preserved Ejection Fraction. J. Am. Heart Assoc..

[10] van der Stam J.A., Bouwmeester S., van Loon S.L.M., van Riel N.A.W., Dekker L.R., Boer A.K., Houthuizen P., Scharnhorst V. (2023). Prognostic Value of Combined Biomarkers in Patients With Heart Failure: The Heartmarker Score. Ann. Lab. Med..

[11] Yancy C.W., Jessup M., Bozkurt B., Butler J., Casey D.E., Drazner M.H., Fonarow G.C., Geraci S.A., Horwich T., Januzzi J.L. (2013). 2013 ACCF/AHA guideline for the management of heart failure: A report of the American College of Cardiology Foundation/American Heart Association Task Force on Practice Guidelines. J. Am. Coll. Cardiol..

[12] Levey A., Stevens L.A., Schmid C.H., Zhang Y.L., Castro A.F., Feldman H.I., Kusek J.W., Eggers P., Van Lente F., Greene T. (2009). A new equation to estimate glomerular filtration rate. Ann. Intern. Med..

[13] Benfari G., Antoine C., Miller W.L., Thapa P., Topilsky Y., Rossi A., Michelena H.I., Pislaru S., Enriquez-Sarano M. (2019). Excess Mortality Associated with Functional Tricuspid Regurgitation Complicating Heart Failure with Reduced Ejection Fraction. Circulation.

[14] Bannehr M., Edlinger C.R., Kahn U., Liebchen J., Okamoto M., Hähnel V., Dworok V., Schipmann F., Kücken T., Bramlage K. (2021). Natural course of tricuspid regurgitation and prognostic implications. Open Heart.

[15] Konstam M.A., Kiernan M.S., Bernstein D., Bozkurt B., Jacob M., Kapur N.K., Kociol R.D., Lewis E.F., Mehra M.R., Pagani F.D. (2018). Evaluation and Management of Right-Sided Heart Failure: A Scientific Statement From the American Heart Association. Circulation.

[16] Kodali S., Hahn R.T., Eleid M.F., Kipperman R., Smith R., Lim D.S., Gray W.A., Narang A., Pislaru S.V., Koulogiannis K. (2021). Feasibility Study of the Transcatheter Valve Repair System for Severe Tricuspid Regurgitation. J. Am. Coll. Cardiol..

[17] Haberle A.D., Biggs M.L., Cushman M., Psaty B.M., Newman A.B., Shlipak M.G., Gottdiener J., Wu C., Gardin J.M., Bansal N. (2021). Level and Change in N-Terminal Pro-B-Type Natriuretic Peptide and Kidney Function and Survival to Age 90. J. Gerontol. Ser. A-Biol. Sci. Med. Sci..

[18] Yu J., Oh P.C., Kim M., Moon J., Park Y.M., Lee K., Suh S.Y., Han S.H., Byun K., Ahn T. (2017). Improved early risk stratification of patients with ST-segment elevation myocardial infarction undergoing primary percutaneous coronary intervention using a combination of serum soluble ST2 and NT-proBNP. PLoS ONE.

[19] Rola P., Miralles-Aguiar F., Argaiz E., Beaubien-Souligny W., Haycock K., Karimov T., Dinh V.A., Spiegel R. (2021). Clinical applications of the venous excess ultrasound (VExUS) score: Conceptual review and case series. Ultrasound J..

[20] Mirna M., Topf A., Wernly B., Rezar R., Paar V., Jung C., Salmhofer H., Kopp K., Hoppe U.C., Schulze P.C. (2020). Novel Biomarkers in Patients with Chronic Kidney Disease: An Analysis of Patients Enrolled in the GCKD-Study. J. Clin. Med..

